# Prejudice, Proxemic Space, and Social Odor: The Representation of the ‘Outsider’ Through an Evolutionary Metaverse Psychology Perspective

**DOI:** 10.3390/brainsci15080779

**Published:** 2025-07-22

**Authors:** Sara Invitto, Francesca Ferraioli, Andrea Schito, Giulia Costanzo, Chiara Lucifora, Assunta Pompili, Carmelo Mario Vicario, Giuseppe Curcio

**Affiliations:** 1 Laboratory on Cognitive and Psychophysiological Olfactory Processes, DiSTeBA, University of Salento, 73100 Lecce, Italy; andrea.schito@unisalento.it; 2 Laboratory of Cognitive and Social Neuroscience, Department of Cognitive Science (COSPECS), University of Messina, 98121 Messina, Italy; francesca.ferraioli@unime.it (F.F.); giulia.costanzo@unime.it (G.C.); carmelomario.vicario@unime.it (C.M.V.); 3 Dipartimento di Filosofia e Comunicazione, Università di Bologna, 40126 Bologna, Italy; chiara.lucifora@unibo.it; 4 Department of Biotechnological and Applied Clinical Science (DISCAB), University of L’Aquila, 67100 L’Aquila, Italy; assunta.pompili@univaq.it (A.P.); giuseppe.curcio@univaq.it (G.C.)

**Keywords:** social bias, prejudice, proxemic behavior, olfactory perception, putative pheromone virtual reality, implicit association test, EEG

## Abstract

Prejudices, particularly those related to social biases, are shaped by various cognitive and sensory mechanisms. This study investigates the interaction between olfactory perception and propensity and implicit or explicit prejudices through three experimental protocols in a metaverse condition. Experiment 1 examines the impact of five different odors on proxemic behavior when interacting with individuals from stigmatized social groups. Experiment 2 integrates electroencephalography (EEG) to analyze the neural correlates of prejudice-related responses to olfactory stimuli. Experiment 3 explores implicit biases through the Implicit Association Test (IAT) in relation to different fragrances, without employing virtual reality. The proposed protocol is expected to demonstrate a significant relationship between olfactory cues, linked to social relationships, and implicit or explicit prejudices, with variations based on individual differences. These insights will contribute to psychological, neuroscientific, and social interventions, offering new perspectives on the unconscious mechanisms of bias formation. Additionally, this study highlights the potential of virtual reality and olfactory stimuli as innovative tools for studying and addressing social biases in controlled environments.

## 1. Introduction

The recent literature suggests that implicit prejudices affect our daily behavior substantially, especially when considering inter-individual interactions in a social context and their effect on disadvantaged people. The presence of implicit biases toward outgroups, minorities, and even women has been well documented [1], including their consequences [2]. An interesting review [3] examining several articles on the most popular gender stereotypes and implicit biases has highlighted how scientific evidence often disproves and challenges them. Furthermore, it has been reported that implicit biases are frequent in healthcare professionals [4,5,6]. Data from these studies indicate that health providers adopt implicit negative attitudes, reflected in a lower quality of care toward individuals belonging to stigmatized groups and therefore favoring health disparities (e.g., between white people and individuals belonging to different ethnicities). A possible explanation for this phenomenon from an evolutionary perspective has been proposed, hypothesizing that the existence of a behavioral immune system (BIS) would have developed initially to avoid exposure to pathogens and parasites [7]. This system is activated by specific morphological stimuli which automatically trigger emotional and cognitive responses such as disgust and automatic inference concerning the signs of potential sickness. However, the BIS is hypersensitive and can misidentify unfamiliar people—such as ethnic minorities, immigrants, or disabled individuals—as threats. This misfiring leads to automatic disgust, social distancing, and implicit prejudice. Sensory cues like body odor, disgust, and proxemics also activate BIS-related biases [8,9]. Indeed, some studies link disgust sensitivity with xenophobia and authoritarian attitudes [10]. In healthcare, such biases contribute to disparities in treatment quality. The BIS thus underlies subtle forms of racism and discrimination [11]. Understanding it helps explain how prejudice can stem from unconscious, biologically rooted reactions. Consequently, stigmatized groups and minorities (e.g., disabled people, immigrants, sexual minorities, etc.) suffer avoidance and social isolation. Morphological features do not seem to be the only stimuli activating the BIS. Probably individuals avoid the ‘outsider’ through other cultural and biological mechanisms. Among the latter, proxemics and olfaction are both related to implicit prejudices. Olfactory perception, which plays a crucial role in evoking disgust-related feelings [8] and detecting pathogen threats [12,13], can contribute to the establishment and maintenance of implicit biases and stereotypes. In fact, the sense of smell represents an essential means of information transfer, crucial for promoting relationships between species [14,15] and within species [16]. Olfactory perception can be modulated and shaped by the sense of social identity and by the belonging social group [17,18].

Moreover, the information processing decoded by human odor signals does not require conscious awareness [19] and is applied to various human relational contexts. Intriguingly, the exposure of male subjects to the putative human pheromone estratetraenol results in an increase in prosocial behavior demonstrations [20].

The BIS and olfaction, as well as proxemics, are supposed to act in a way that allows one to perceive threatening stimuli and consequently adopt physiological and behavioral defense strategies. It ensures the maintenance of a margin of safety between the individual and the surrounding world [21]. The invasion of personal space is not only caused by physical factors (e.g., physical contact with humans or objects) but also caused by other sensory factors such as noise, smells, or unwanted eye contact [22].

Recent findings suggest that proxemic behavior parameters change according to the interacting agent’s features, such as ethnicity [23] and sexual orientation [24]. An interesting study [25] found that Caucasian people embodied in a black body in virtual reality (VR) reduced their implicit bias after cooperating with a black avatar in the VR environment. Additional research indicates that the perception of threat can alter how individuals judge spatial distances to out-group members, leading to an increased perceived distance [26]. This suggests that prejudices and biases are reflected in spatial perception, reinforcing social boundaries and avoidance behaviors. These findings highlight how social biases manifest even in virtual environments, shaping interpersonal distance and reinforcing implicit attitudes. VR systems are now more frequently employed in experimental psychology [27], revealing their essential contribution in several processes such as analyzing social dynamics [28], affective processing [29,30], and spatial representation [31], as well as empowering clinical treatments [32,33,34]. For instance, recent findings highlight the efficacy of virtual reality exposure therapy (VRET) in therapy that targets fear-of-contamination disgust [34].

Although the literature shows the relevance and reciprocal influence between olfaction, proxemic behavior and implicit prejudice, and exploiting virtual environments, their relationship is still far from being understood. Although there is much debate about whether human pheromones are poorly perceived due to the absence of the human vomer nasal organ [35,36,37], many experimental studies using putative pheromones in humans indicate that they affect social behavior both in the simple behavioral field [37,38,39,40] and in the fields of communication media [41] and robotics [42], even analyzing these aspects from an electrophysiological point of view [38,41,43]. Moreover, male and female axillary secretions (MASs and FASs) are recognized as significant components of human body odor and have been implicated in chemosensory communication. Several studies have reported hormonal and behavioral responses to MAS and FAS extracts [44,45,46]; however, whether these compounds meet the current criteria for classification as human pheromones remains a subject of ongoing debate. The neural mechanisms through which such cues might influence social cognition—particularly proxemics and prejudice, both implicit and explicit—are poorly understood, especially in ecologically valid contexts such as those enabled by virtual reality. Indeed, the connection between olfaction, proxemics, and implicit prejudice lies in the subtle sensory cues that unconsciously shape social judgments. Olfaction, especially sensitivity to body odor, has been shown to activate disgust responses, which are tied to the BIS and can trigger social avoidance. Similarly, proxemic behavior, can reflect unconscious evaluations of threat or discomfort, often linked to perceived “otherness.” Individuals from stigmatized groups may be unconsciously perceived as less familiar or safe, prompting both increased physical distance and negative emotional responses. These reactions, although automatic, can reinforce implicit biases, particularly when cultural or sensory unfamiliarity is misinterpreted as a potential dysfunctional cue. In turn, implicit prejudice can further condition sensory perception (e.g., interpreting neutral smells as unpleasant when associated with an outgroup). Virtual environments, though ideal for controlled testing, may allow the multisensory depth of real-world interactions, making the environment more ‘ecological’ [47].

As far as it is known, no research work has been conducted for the investigation of prejudices and/or gender stereotypes during electroencephalographic (EEG) recordings and the administration of social odors (putative human pheromones), also taking into account proxemic behavior parameters. The EEG is a valuable and powerful non-invasive tool for studying brain activity in experimental contexts. It has also been used for the analysis of event-related olfactory potentials (OERPs) [48,49] and has proven helpful in protocols that employed subthreshold olfactory stimuli [50], such as putative human pheromones [41,51]. Furthermore, the electroencephalographic signal appears sensitive in discriminating such molecules [52] and differentiating between molecules with similar characteristics [53]. A recent study [41] found that putative human pheromones affected subjects’ EEG signal and the sense of co-presence in a gender-dependent fashion. Thus, EEG would be helpful to achieve a deeper understanding of the neural mechanisms underlying prejudice, its relationship with olfactory aspects, and possible strategies that can be used to overcome the implicit fear of the “outsider”.

Some studies have shown that prejudice can be based on and is associated with religious beliefs [54,55]. Indeed, the association of religiosity with racial and sexual prejudice has well and long been known, with more religious people being more prejudiced [56]. For example, Tsang and Rowatt [57] have shown that negative attitudes towards homosexual individuals, measured with the Implicit Association Test (IAT) [58], were predicted by intrinsic religious orientation, a form of religiosity that has an end in itself [59]. Recent evidence suggests that both intrinsic and extrinsic religiosity (the latter being a form of religiosity instrumental to obtain some benefits, such as status) are associated with prejudice. In contrast, agnosticism is associated with reduced prejudice and greater tolerance [60]. In our framework, religion is not treated as a target of bias but as a factor that may modulate prejudice expression, particularly in interaction with olfactory cues.

### 1.1. Experimental Hypotheses

Based on this research, we hypothesize that individual factors such as odor perception, disgust sensitivity, and religiosity influence explicit and implicit prejudices towards minorities. We predict that odors associated with disgust will modulate proxemic behavior and prejudice-related responses in a VR scenario and at IAT. The use of VR is expected to offer great ecological validity. Furthermore, integrating EEG indices in association with manipulated prejudice and fragrances can help better characterize the investigated phenomenon’s electrophysiological dynamics.

In particular, we want to test the following three hypotheses in three different experiments:

**Hypothesis** **1.** *Experiment 1 investigates the influence of different types of odors on proxemic behavior when interacting with individuals from various discriminated social categories. Additionally, this experiment will assess how individual traits, such as disgust sensitivity, social odor perception, and religiosity, affect participant behavior in these interactions. We hypothesize that longer reaction times (RTs) reflect stronger implicit prejudice, as greater cognitive-emotional conflict arises when individuals interact with out-group avatars. Previous studies have shown that implicit bias can delay behavioral responses [61] and that prejudiced individuals are more likely to perceive out-group faces as threatening, which increases processing time [62]*.

**Hypothesis** **2.** *Experiment 2 explores the effects of different types of odors on proxemic behavior when interacting with individuals from various discriminated social categories, from behavioral and electrophysiological perspectives. We hypothesize to find different pathways of cortical activation, evidenced through the analysis of EEG rhythms and components elicited by the choice of position in VR. In particular, we expect that the conditions connected to the presentation of the social odor are evidenced by gender-related electrophysiological responses. We hypothesize that exposure to socially connoted odors will elicit distinct patterns of cortical activation, particularly in frontal and parietal areas involved in social cognition, emotional evaluation, and spatial attention. Specifically, alpha (8–12 Hz) and theta (4–7 Hz) band power changes are expected to reflect cognitive and emotional engagement [63], while mu rhythm suppression (8–13 Hz over sensorimotor cortex) may index embodied simulation, in social interaction [64], social victimization [65] or empathic mirroring [66]. Indeed, previous neuroimaging research with adults highlighted greater empathic responses to racial in- vs. outgroups across a variety of paradigms in EEG using mu suppression [67]. The last hypothesis is connected to gender-related EEG responses: we expect the gender-specific modulation of EEG signals, with male and female participants showing differential activation patterns depending on the congruence between the odor and the perceived gender of the avatar (e.g., greater frontal theta or parietal alpha desynchronization may be observed in incongruent odor–gender pairings, reflecting increased cognitive load or ambiguity resolution) [68,69]*.

**Hypothesis** **3.** *Experiment 3 will use a behavioral approach to investigate the influence of different odors on implicit association when interacting with individuals from various discriminated social categories. Again, disgust sensitivity, social odor perception, and religiosity will be considered. At first, we expect to observe significant changes in the IAT index (see below for information on how it is calculated), which can confirm the implicit association between positive/negative perception and some specific social categories (ethnicity, sexual orientation, body shape) based on cultural prejudice. Moreover, we expect this effect to be modulated by fragrances. More specifically, with respect to control fragrance (Vaseline), male/female hormones would influence the perception of pleasantness, mainly of sexual orientation stimuli, and sweat odor would influence mainly the perception of stimuli characterized by different ethnicity and body shape.*

### 1.2. Aim

Considering the presented evidence, this study protocol aims to promote a new methodology for analyzing the influence of odors on our behavior and believes that exploit virtual environments and implicit perception. To do so, we aim to develop an experimental protocol to analyze the effects of odors associated with religiosity and social odor on explicit and implicit prejudice, through virtual reality and behavioral measurements.

To achieve this, we have designed three experimental studies to be conducted at three different universities departments: the Department COSPECS, from the University of Messina, the Department of Biological and Environmental Sciences and Technologies (DiSTeBA), University of Salento, and the Department DISCAB, University of L’Aquila.

The first study conducted at the University of Messina, Experiment 1, examines the impact of five different types of odors on proxemic behavior when interacting with individuals from various discriminated social categories. Additionally, this experiment will assess how individual traits, such as disgust sensitivity, social odor perception, and religiosity, affect participant behavior in these interactions. We expect that participants will more frequently choose to sit away from stigmatized avatars (e.g., homosexual, obese, or foreign couples), particularly under unpleasant odors like sweat. Neutral or pleasant odors may attenuate this effect. Avoidance behavior is thus expected to vary as a function of both odor condition and avatar type. Moreover, individuals high in disgust sensitivity or religiosity are expected to show stronger avoidance across conditions.

The second study, Experiment 2, conducted at the University of Salento, expands upon Experiment 1 by integrating EEG measurements into the research protocol. This will allow for a deeper exploration of the neural correlates underlying prejudice-related responses and the effects of odors on brain activity.

The third study, Experiment 3, will not utilize VR like the previous two but will assess implicit prejudice responses using the IAT in relation to different fragrances. This approach will provide additional insights into how olfactory cues influence implicit biases in a non-immersive and more ecological setting.

By conducting these three studies, we aim to comprehensively analyze the interaction between olfactory stimuli, individual predispositions, and social biases, contributing to a deeper understanding of how prejudices manifest in both virtual and real-world contexts. By combining EEG with VR-based behavioral tracking, this experiment bridges the gap between affective neuroscience and social psychology. Social odors may not only bias spatial behavior but also elicit measurable changes in neural dynamics, reflecting altered perception, attention, and embodiment during intergroup interaction.

## 2. Materials and Methods

### 2.1. Experimental Protocol

The whole experimental protocol includes three different experimental studies in which behavioral and electrophysiological measurements will be acquired while manipulating fragrances and social biases through VR scenarios (Experiment 1 and 2) or measuring it through IAT (Experiment 3).

In Experiment 1 and 2, the VR session consists of a waiting area of a virtual subway where participants have to choose where to sit, that is, sitting near to a standard or non-romantic couple, instead of a homosexual, foreign, or obese one, with respect to the three different conditions, presented in counterbalanced order. Participants have to repeat the VR task in five different odor conditions, also presented in counterbalanced order, which are neutral odor (i.e., Vaseline oil), incense, sweat, and male and female putative pheromones. For Experiment 3, a dedicated computerized IAT protocol was developed: in this case, participants were asked to associate words and pictures related to different discriminated social categories under the same five odor conditions listed above.

### 2.2. Olfactory Stimuli

Subjects will be tested on 5 odorant conditions. These conditions will be referred to as follows: for a neutral substance Vaseline oil will be used, for social odors putative pheromones will be used, for unpleasant odor sweat will be used, and for pleasant odor incense will be used. In particular, Vaseline oil (N) will be used as a control substance [70,71], propionic acid (CAS-No: 79-09-4) as a frankincense-like odor (P), ursolic acid (CAS-No:77-52-1) for sweat odor (U), and 5α-Androst-16-en-3α-ol (CAS-No:1153-51-1) and Equilin (CAS-No:474-86-2) as male (A) and female putative pheromones (E), respectively. All of them, unless Vaseline oil, are provided by Merck KGaA (Darmstadt, Germany). Except N and P, which are available in a ready-to-use formulation, U, A, and E will be dissolved in Vaseline oil in a 1mg:1ml proportion. Then, each solution will be centrifuged for 5 min, mixed by vortex, and then stored in a glass vial (Agilent Technologies, Inc., Santa Clara, CA, USA). Before the experimental session, five plastic food containers (i.e., one for each odor condition) will be prepared and labeled. Make-up remover pads will be cut in half and some of the halves will be moistened with 4 drops of a specific substance and kept closed in the corresponding container. One drop will be used for P, due to its intensity. Each drop will be delivered using a 10 mL syringe. During the experiment, the olfactory stimuli will be administered using a headband microphone mounted on the subject’s head. The half pad for each odor condition will be placed on the top of the microphone by an experimenter equipped with nitrile gloves. It is specified that it is not a task where a sensorial/perceptive response to odors is measured, nor is an olfactometer used. It is a procedure where, as in previous studies carried out [41,42], we use the olfactory stimulus as a modulator of a cognitive/behavioral response

### 2.3. Participants

All participants will be recruited via online platforms and presentations at Psychology courses at Messina University, University of Salento, and University of L’Aquila. Participants for each unit (i.e., 93 for each experiment; range age 18-40) will be evaluated based on general anamnesis data and olfactory psychophysical response. The ortho-nasal olfactory function will be assessed using the validated Sniffin’ Sticks-16 test battery (Burghart Messtechnik, Wedel, Germany). Participants will provide informed consent, and this study will be conducted according to the department’s ethical policies, with protocol submitted to the local Institutional Review Board (Ethical approval protocol number: Protocol Code n.3/2024 IRB DiSTeBA). Subjects with diagnosed neurological, psychiatric, diabetes, respiratory, or ENT disorders will be excluded from the experiment. Moreover, participants undergoing pharmacological or hormonal treatment (including the contraceptive pill) will be excluded from this study. Subjects who are hyposmic or anosmic when evaluated with the Sniffing Stick test will also be excluded from the experiment. To define the sample size, an analysis was conducted by first evaluating the literature present in Larson & Carbine’s review, which indicates that in EEG the value of the effect size is almost never reported in the EEG literature, and of the two works analyzed, in the meta-analysis, only two are of an olfactory type [72]. In any case, we referred to the theoretical and expected eta squared of 0.08. To define the sample size, an a priori analysis was performed on Jamovi 2.6.44 with PAMLj extension setting the following indications: General Linear Model Power Analysis, between-subjects factor 1 (3 conditions) and factor 2 (5 conditions); parameters: minimum power expected, 0.8, α: 0.05; model degree of freedom, 5; expected pη^2^, 0.08. N estimated 93 subjects as necessary to have an f^2^ of 0.0870 and a power of 0.8.

### 2.4. Data Collection Procedure and Study Assessment

Concerning Experiment 1, upon enrollment into this study, participants will be asked to assess olfactory and disgust perception; also, personal beliefs and demographic information will be collected, such as information on nationality, religiosity, and political and sexual orientations. Ortho-nasal olfactory function will be assessed using the validated Sniffin’ Sticks-16 test battery (Burghart Messtechnik, Wedel, Germany) including phenylethyl-alcohol (PEA) odor thresholds, discrimination, and identification. Moreover, participants will be asked to complete the Disgust Propensity and Sensitivity Scale-Revised (DPSS-R) (the Italian version validated by [73]), the Body Odor Disgust Scale (BODS) (the Italian version validated by [74]), and the Religious Attitude Scale (RAS) (the Italian version validated by [75]). In Experiment 2, participants will be assessed through the Sniffin’ Sticks-16 test. Next, the subjects will be recorded via a high-density electroencephalogram. The electroencephalographic (EEG) signals will be acquired from participants’ scalps using a 64-active-electrode cap (ActiCHamp, Brain Products, Munich, Germany), following the international 10–10 system, with a 1000 Hz sampling rate. Electrodes placed above and below the left eye, as well as above the right eye, will be used to track eye movements. The reference electrode will be positioned at FCz, with the signal being offline-referenced to the mastoid electrodes. Impedance levels will be maintained below 5 k.). After the EEG montage a headset microphone will be positioned in a central position of the nose and will be set up to position olfactory stimuli. Afterwards, participants will be asked to complete a questionnaire via Google Forms on their mobile device. The questionnaire includes the following scales: the SOS [76], the BODS [74], and the RAS [75]. Afterward, in parallel with EEG recordings, the participants will wear a Meta Quest 3 virtual reality headset and be placed in a “baseline” scenario, where they can complete a preliminary trial and receive task instructions. To minimize olfactory and carryover effects, inter-trial intervals (ITI) will range from 40 to 60 s to minimize olfactory adaptation and carryover effects, with a random jitter of ±5 s [77,78,79]. In our experiment, due to the time necessary to change the odorant, we will use this interval between conditions. Residual odor traces in the VR headset will be mitigated using a combination of odorless air flushing and non-scented surface cleaning between trials. Also, in Experiment 3 the enrollment phase will be aimed at assessing olfactory and disgust perception, as well as personal beliefs and demographics (nationality, religiosity, and political and sexual orientations). Olfactory function will also be assessed using the validated Sniffin’ Sticks-16 test battery (Burghart Messtechnik, Wedel, Germany) including phenylethyl-alcohol (PEA) odor thresholds, discrimination, and identification. Moreover, participants will be asked to complete the DPSS-R [80] (the Italian version validated by [73]), the BODS (the Italian version validated by [74]), and the RAS (the Italian version validated by [75]).

### 2.5. Experimental Designs

#### 2.5.1. Experiment 1: The Influence of Social Odors on Prejudice in the Metaverse

**Figure 1:**
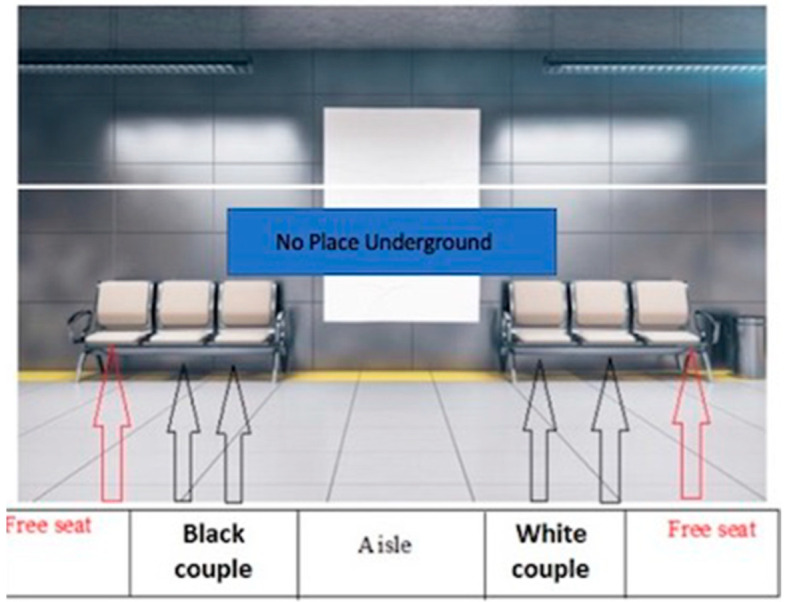
Virtual underground. An example of the scenario with free and occupied seats in the subway stops conditions of racial bias.

**Figure 2:**
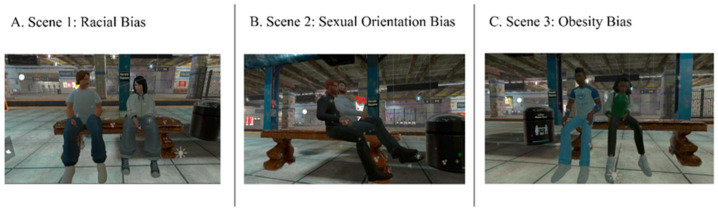
Virtual characters. The avatar couples used for the manipulation of social biases. (**A**) **Scene** 1: Racial Bias; (**B**) Scene 2: Sexual Orientation Bias; (**C**) Scene 3: Obesity Bias.

To study the influence of different scents on explicit prejudice, we will observe participants’ behavior about their interaction with different stigmatized social groups. Participants will be placed within a VR environment reproducing that of a subway (see Figure 1); their task will be to choose if to sit on the bench next to one of the three groups represented in Figure 2A–C, relating to the different social biases examined, or in another place next to standard couple. The participant selection, that is, the side of the bench chosen to sit, right or left, and the reaction time, that is, the time elapsed between the presentation of the VR scene and the moment in which the participant expresses their choice by heading towards the bench to sit on, will be measured. The RT acquisition will be performed in real time during the whole experimental session, by using a custom time decoder in Matlab, which calculates the time elapsed between the experimenter pressing two keys on the keyboard, indicating the time window described above, for each participant’s selection.

The experimental design is a within-subject design where participants repeat the seating place selection for each of the three social biases (i.e., racial, homosexual, and aesthetic bias) and the five odor conditions (i.e., N, P, U, A, and E), in a counterbalanced way, for a total of 15 trails per subject.

During the seat selection, individuals will be exposed to the five odors conditions as described in the previous section. According to previous evidence documenting higher implicit prejudice toward homosexuality in religious individuals [29]. Since body odor disgust sensitivity was found to be associated with xenophobia and prejudice [8], we also expect a similar effect after the exposure to the odor of sweat and that results for both scents might correlate with the DPSS-R [73,80] and BODS [74].

#### 2.5.2. Experiment 2: Neural Correlates of Social Odor Bias in the Metaverse

Experiment 2 integrates the study of electrophysiological activity into the same protocol used in Experiment 1. As previously stated, the VR task and the RTs collection procedure remain identical. Putative human pheromones and social odors will be administered to influence both social behavior [42,81] and electrophysiological activation (measured in resting state conditions, with a baseline neuter baseline condition of 30 sec., and experimental conditions through EEG rhythms) [41,82,83,84]. In line with these findings, we expect that at least one of the task’s factors (i.e., social group or social odor conditions) will affect participants’ choices at both behavioral and electrophysiological levels. For instance, participants may be more likely to choose to sit next to a standard couple than a couple belonging to a stigmatized group. Furthermore, differences in the type of choice and in reaction times (RTs) are expected depending on the type of couple selected (i.e., standard vs. stigmatized) and on the odor condition. We also anticipate that both the experimental conditions and the nature of the choice will significantly influence EEG signals.

#### 2.5.3. Experiment 3: The Role of Social Odors in Implicit Association Related to Prejudice

Experiment 3 aims at studying the influence of different types of odors on implicit association when interacting with individuals from various social categories. Participants will be asked to complete an ad hoc developed Implicit Association Test (IAT); the IAT is a psychological assessment designed to measure the strength of automatic (implicit) associations between concepts in a person’s mind, often revealing unconscious biases [85]. In the typical IAT protocol, participants are asked to quickly categorize two sets of concepts (like “young” vs. “old” or “male” vs. “female”) and two sets of attributes (like “good” vs. “bad”) by pressing specific keys; congruent/compatible (e.g., young–good) and incongruent/incompatible pairs (e.g., old–good) continuously alternate; faster reaction times for certain pairings suggest stronger implicit associations. The present version of the IAT follows the classical procedure articulated in a series of different tasks [86] and aims to investigate potential biases related to particular social groups characterized by different ethnicity (black vs. white individuals), sexual orientation (heterosexual vs. homosexual), and body shape (obese vs. lean individuals). For each of the aforementioned categories, ad hoc images were created using an artificial intelligence system (Midjourney). Moreover, words with pleasant and unpleasant valence were selected from the ANEW database [87] (Montefinese et al., 2014) to be associated with the images. The words from the ANEW database have been selected on the basis of valence, frequency of use (in the written language), and length (in number of letters). A list of ten positive/pleasant and ten negative/unpleasant words (basically adjectives) has been extracted, and the selection was based on scores of valence (high vs. low) and on a comparable frequency of use in the written language. Thus, the positive ones are characterized by high pleasantness (M = 8.07±1.25) while the negative ones have a low pleasantness (M = 2.23±1.32). Positive/pleasant and negative/unpleasant words also showed a comparable frequency of use (LnColfis equal to 4.63 and 3.04, respectively). Finally, the length was virtually identical: 7.7 for negative and 7.6 for positive ones. Table 1 lists the selected words (and the English equivalent).

The whole protocol was developed following the gold-standard procedures published in the literature [88].

### 2.6. Data Analyses

In Experiment 1, all statistical analyses will be conducted using customized R and Matlab scripts, or JASP. Prior to inferential testing, data will undergo preliminary checks to assess assumptions of normality and homogeneity of variance. The normality of continuous variables (e.g., RT, questionnaire scores) will be examined using the Shapiro–Wilk or Kolmogorov–Smirnov test, complemented by visual inspections of histograms and Q-Q plots. Homogeneity of variance across groups will be verified using Levene’s test. To analyze seating choice (binary categorical variable left vs. right), a Chi-square test of independence (χ^2^) will first be performed to assess whether seat selection is associated with olfactory stimulation conditions, avatar characteristics, or questionnaire scores (categorized if necessary). Additionally, a binary logistic regression model will be implemented to predict seating choice based on olfactory stimulation type, avatar characteristics, and individual differences (e.g., religiosity, disgust sensitivity). For RT, repeated-measures ANOVA will be conducted to compare reaction times across different olfactory stimulation conditions and avatar characteristics. A Friedman test (non-parametric equivalent) will be used if the normality assumption is violated. A multiple linear regression model will be applied to examine whether sensory stimulation, questionnaire scores, or demographic variables significantly predict reaction time. Pearson or Spearman correlation analyses will be used to explore relationships between questionnaire scores and behavioral measures (seating choice, reaction time). Finally, mediation and moderation analyses will be conducted to determine whether disgust sensitivity or religiosity mediates the effect of sensory stimulation on seating choice. Moderation analyses will assess whether the relationship between sensory stimulation and behavior varies as a function of individual differences (e.g., religiosity moderating the effect of odor on seat choice). To account for potential demographic confounds, age, gender, and education level will be included as covariates in relevant models. Exploratory interaction effects will also be tested using factorial ANOVA to determine whether specific combinations of sensory stimuli and avatar characteristics influence behavioral outcomes. Statistical significance will be set at *p* < 0.05, and effect sizes (e.g., Cohen’s d, partial eta squared) will be reported where applicable. In Experiment 2 the same analysis as in Experiment 1 will be conducted on the behavioral data by using JASP and Matlab. Instead, the EEG recording sessions will be pre-processed and analyzed mainly using the BrainVision Analyzer 2.3.0 software (Brain Products GmbH, Gilching, Germany). The neural tracks will be aligned to the RTs timestamps for the rhythms. This alignment strategy allows us to compare neural responses associated with proxemic decisions across different olfactory conditions. EEG Preprocessing: EEG preprocessing and analysis will be performed according to the recent literature on the time–frequency analysis of olfactory and chemosensory stimuli in EEG [89]. The system to analyze the EEG tracks will be BrainVision Analyzer (Brain Products, Germany). EEG data will be re-referenced to the average of the left (M1) and right (M2) mastoids. To reduce slow drifts and high-frequency noise, data will be band-pass filtered using a zero-phase Butterworth filter with a frequency range of 0.3 to 30 Hz. Following filtering, continuous EEG data will be segmented into 2.0-s epochs ranging from −0.5 s to +1.5 s relative to stimulus onset. A baseline correction will be applied using a pre-stimulus interval from −0.5 s to 0 s. Independent Component Analysis (ICA) will be performed to correct ocular artifacts. Components representing ocular artifacts (e.g., eye blinks or movements), identified by their time course and frontal topography, will be manually rejected. Epochs containing amplitude values exceeding ±100 µV will be excluded from further analysis, as such values will likely reflect EEG artifacts. A time-frequency (TF) analysis will be performed using the continuous Morlet wavelet transform (CWT) to assess oscillatory dynamics. This method allows for the decomposition of EEG signals into time-resolved frequency components. The Morlet wavelet, consisting of a complex exponential modulated by a Gaussian envelope, will compute amplitude across time and frequency dimensions. All preprocessing steps and criteria for artifact rejection are determined a priori and will be applied uniformly across all participants.

In Experiment 3 reaction times will be the main dependent variable, and a IAT index will be calculated as the difference between the speed of responses in different conditions (congruent/easy and incongruent/difficult). For such an index, repeated-measures ANOVA will be conducted to compare reaction times across different olfactory stimulation conditions and avatar characteristics. A Friedman test (non-parametric equivalent) will be used if the normality assumption is violated. A multiple linear regression model will be applied to examine whether sensory stimulation, questionnaire scores, or demographic variables significantly predict reaction time. Finally, ad hoc analyses will be conducted to determine whether disgust sensitivity or religiosity mediates the effect of sensory stimulation. To account for potential demographic confounds, age, gender, and education level will be included as covariates in relevant models. Statistical significance will be set at *p* < 0.05, and effect sizes (e.g., Cohen’s d, partial eta squared) will be reported where applicable.

## 3. Discussion

This research aims to explore the relationship between disgust and prejudice by investigating the relationship between disgust sensitivity, odor perception, and implicit prejudices through three experimental protocols conducted across different universities. Experiment 1 examines the influence of five different types of odors on proxemic behavior when interacting with individuals from various discriminated social categories. Additionally, it evaluates the role of individual traits, such as disgust subjective sensitivity, social odor perception, and religiosity, in shaping participant behaviors. Experiment 2, conducted at the University of Salento, builds upon the first study by incorporating EEG measures to assess the neural correlates of prejudice-related responses and the effects of odors on brain activity. Experiment 3, instead of utilizing virtual reality (VR) as the first two studies, assesses implicit prejudice responses using the Implicit Association Test (IAT) to different odorants. Together, these studies offer a comprehensive analysis of the interaction between olfactory stimuli, individual predispositions, and social biases, contributing to a deeper understanding of how prejudices manifest in both virtual and real-world contexts. Discrepancies between implicit and explicit measures—such as IAT scores and proxemic behaviors—will be explored as theoretically meaningful patterns. These may reflect the influence of contextual cues (e.g., social odors) on embodied responses versus stable automatic associations captured by the IAT [90].

Utilizing the metaverse will enable us to assess significant differences between explicit and implicit prejudice, as well as variations with respect to individuals’ differences. We anticipate that these studies will provide deeper insights into both explicit and implicit prejudice and aid in the creation of a neurocognitively plausible computational model of neural circuits. This model will contribute to the development of an evolutionary-based framework that integrates social odor and prejudice within the metaverse. The results generated by the present project will have multiple straightforward implications with a positive impact on the scientific advancement of knowledge about psychology and the psychophysiology of olfactory perception and its contribution, from a psychological, biological, and evolutionary perspective, to the establishment and to the eventual modification of implicit and explicit prejudices in the human mind. Many studies demonstrated that, although odor-induced disgust plays a marginal role in the most widely used disgust sensitivity scales [91], the sense of smell is closely related to this emotion [92]. The emotional expression associated with disgust mimics the expulsion of presumably unpleasant substances from the mouth [93], and nose wrinkling minimizes air intake through the nose [94]. While taste, as noted by Rozin et al. [95], primarily triggers disgust to prevent the ingestion of harmful substances, smell, according to Stevenson [12], offers an early detection system for microbial threats. A disgusting odor can alert us to dangers such as spoilage or toxic substances before they come into direct contact with our body. Together, these chemical senses form a comprehensive defense mechanism, highlighting the intricate ways in which humans avoid environmental threats to maintain health.

There is research suggesting that disgust plays an important role in religiosity. Religiosity refers to the degree of an individual’s religious beliefs, behaviors, and practices, as well as the influence of religion on their daily life and social interactions. It encompasses both personal and social aspects of religion and can vary widely between individuals, cultures, and religious traditions. It is important to underline the differences between religiosity and spirituality. According to Miller and Thoresen [96], spirituality corresponds to a phenomenon tied to the individual, whereas religiosity is more closely linked to social aspects. For MacDonald [97], religiosity is one of the aspects of spirituality; the others include cognitive orientation toward spirituality, the experiential/phenomenological dimension, existential well-being, paranormal beliefs, and religiosity itself. The author also identifies other types of religiosities, such as religious affiliation, attendance at religious places, and religious motivation.

Some researchers found a positive and significant correlation between disgust sensitivity and general religious fundamentalism [98,99,100], as well as on specific forms of religious scrupulosity [101,102]. Although it appears that disgust may facilitate religiosity, some researchers have highlighted the role of religion as an adaptive response to environmental threats associated with feelings of disgust. According to the evolutionary theory detailed by Tybur et al. [103], there are three domains of disgust sensitivity—three groups of situations or contexts in which the basic emotion of disgust functions to facilitate individual, cultural, and evolutionary adaptation, promoting survival and reproductive success. This specific model of disgust consists of pathogenic, sexual, and moral domains of disgust sensitivity [100,101].

The expected results of this research include a significant correlation between odors associated with disgust and increased prejudice-related behaviors. We anticipate that participants exposed to odors related to sweat and other bodily emissions will show stronger implicit biases compared to those exposed to neutral or pleasant odors. We also expect that individuals with higher disgust sensitivity and religiosity will demonstrate stronger prejudice tendencies in both explicit and implicit assessments. The EEG data in Experiment 2 should provide further insight into the neural mechanisms underlying these associations, potentially revealing heightened neural responses to social outgroups in participants with stronger disgust sensitivity. Experiment 3 is expected to complement these findings by confirming that implicit biases are modulated by olfactory cues even outside of immersive VR environments.

### 3.1. Study Limitation

An important limitation not addressed in the current discussion is the potential for demand characteristics in immersive VR settings. Given the rich sensory context and the repeated exposure to odor cues, participants may infer this study’s aims, potentially biasing their behavioral or neural responses. To mitigate this, future studies could incorporate cover tasks or assess participant awareness of study hypotheses post hoc to control for expectancy effects. Additionally, the cross-cultural generalizability of olfactory stimuli warrants attention. Odor perception is deeply influenced by cultural background, prior experience, and linguistic associations. The use of specific odorants without considering participants’ cultural context may limit the external validity of the findings (but this aspect in this study is valid only for frankincense-like odor). Indeed, when using putative human pheromones (e.g., androstadienone, estratetraenol), the cultural influence on odor interpretation is arguably reduced, since these compounds are thought to elicit subconscious, biologically driven effects rather than responses based on learned or symbolic odor associations (like food or perfume scents). In the latter case, however, neuroendocrine and hormonal variables may modulate the response to the odorant. In fact, for example, the menstrual cycle can modulate the response to odor and/or pheromones. Compounds such as androstadienone and estratetraenol are believed to act via chemosensory pathways that influence affective or social processing non-consciously, rather than through culturally learned associations. As such, the risk of cross-cultural variability in subjective interpretation is minimized, since the effects of these compounds are thought to be more biologically universal. Therefore, while cultural generalizability may be less of a concern, future work should still consider including physiological or hormonal profiling (e.g., menstrual cycle phase, olfactory receptor genotyping) to better account for inter-individual variability in responsiveness.

### 3.2. Relevance of the Research and Future Perspectives

This research has significant implications for multiple scientific and social domains. By integrating psychological, neuroscientific, and olfactory research within an experimental VR framework, our study contributes to a more comprehensive understanding of prejudice formation and the role of sensory perception in shaping social attitudes. Furthermore, the use of VR and EEG in conjunction with olfactory stimuli offers an innovative approach to examining unconscious biases in ecologically valid settings. From a broader perspective, this study has potential applications in clinical and social interventions. By identifying how disgust sensitivity and olfactory cues influence prejudice, this research may inform strategies for reducing bias through targeted exposure therapy or desensitization techniques. Additionally, this study highlights the necessity of considering sensory modalities in diversity training and inclusion initiatives, as implicit biases are often shaped by non-verbal and subconscious processes. Future research should explore additional sensory dimensions and their influence on social behaviors, including the role of auditory and tactile stimuli in prejudice formation. Furthermore, the integration of neuroimaging techniques, such as fMRI or high-density EEG with better spatial resolution, could provide complementary insights into the neural representation of social biases. Investigating how olfactory perception varies across cultures and different social contexts may also contribute to a deeper understanding of how biases are learned and reinforced. Ultimately, this research represents a crucial step toward developing more effective interventions to mitigate the negative impact of prejudice in diverse social environments.

## Figures and Tables

**Table 1:** List of the selected words from ANEW database.

Positive/Pleasant	Negative/Unpleasant
FELICE (happy)	DEPRESSO (depressed)
AMATO (loved)	SLEALE (disloyal)
LIBERO (free)	SGRADEVOLE (nasty)
ACCOGLIENTE (cozy)	STRESSATO (distressed)
ONESTO (honest)	PERDENTE (loser)
FORTUNATO (lucky)	EGOISTA (selfish)
SOCIEVOLE (social)	SCONFITTO (defeated)
BELLO (beautiful)	OSTILE (hostile)
RISPETTOSO (respectful)	RESPINTO (rejected)
SIMPATICO (nice)	BRUTTO (ugly)

## Data Availability

Data from this research will be available upon request to the corresponding author.
